# Design and Implementation of a Novel Interferometric Microwave Radiometer for Human Body Temperature Measurement

**DOI:** 10.3390/s21051619

**Published:** 2021-02-25

**Authors:** Guangmin Sun, Pan Ma, Jie Liu, Chong Shi, Jingyan Ma, Li Peng

**Affiliations:** Faculty of Information Technology, Beijing University of Technology, Beijing 100124, China; gmsun@bjut.edu.cn (G.S.); mp1001@emails.bjut.edu.cn (P.M.); shic@emails.bjut.edu.cn (C.S.); majy0405@emails.bjut.edu.cn (J.M.); PengLi@emails.bjut.edu.cn (L.P.)

**Keywords:** interferometric, microwave radiometer, correction algorithm, calibration, inversion algorithm, neural network

## Abstract

In this paper, the key technology of interferometric microwave thermometer is studied, the research can be applied to the temperature measurement of human body and subcutaneous tissue. This paper proposes a hardware architecture of interferometric microwave thermometer with 2 GHz bandwidth, in which the phase shifter is used to correct phase error and the quadrature demodulator is used to realize autocorrelation detection function. The results show that when input power is 7 dBm, the detection sensitivity can reach 176.54 mV/dBm and the temperature resolution of the microwave radiometer can reach 0.4 K. Correction algorithm is designed to improve the accuracy of temperature measurement. After correction, the phase error is reduced from 40° to 1.4° and when temperature changes 0.1 °C, the voltage value changes obviously. Step-by-step calibration and overall calibration are used to calibrate the device. Inversion algorithm can determine the relationship between physical temperature and output voltage. The mean square error of water temperature inversion by multiple linear regression algorithm is 0.607 and that of BP neural network algorithm is 0.334. The inversion accuracy can be improved by reducing the temperature range. Our work provides a promising realization of accurate, rapid and non-contact detection device of human body temperature.

## 1. Introduction

The research and development of non-contact temperature measurement equipment is of great significance for medical clinical applications, especially in emergency departments and intensive care units (ICU). The non-contact temperature measurement equipment can speed up the emergency triage process and gain more treatment time; it can measure the body temperature of a person wearing clothes; it can monitor the body surface temperature and subcutaneous tissue temperature of ICU patients within an appropriate distance; it can make a positive contribution to epidemic prevention and control of COVID-19.

At present, people mainly use infrared detection technology for non-contact rapid measurement of human body temperature. The advantages of infrared detection technology are: (1) Non-contact measurement; (2) wide measurement range; (3) high temperature measurement speed; and (4) high sensitivity. However, the infrared detection technology cannot achieve “penetrating” detection, long-distance detection, and high-precision detection, in addition, the performance of detection products is related to the environment. Currently, infrared thermometer is widely used in large stations, shopping malls, etc. as a rough temperature screening.

The research and development of thermometers that can measure temperature through clothing, the temperature measurement accuracy is high and the impact of environment is small, has become the development trend of non-contact temperature measurement equipment.

In microwave band, the microwave attenuation of clothing is small, the influence of ambient light source on microwave is small, the emissivity difference between human body and other objects are large and the radiation energy of human body has a good linear relationship with physical temperature. Furthermore, microwave can penetrate into subcutaneous tissue for temperature measurement.

The purpose of this project is to design a novel non-contact microwave thermometer radiometer and improve its temperature measurement accuracy. This paper studies the hardware structure of the microwave radiometer, in which the complex correlator is the core, the design of software correction algorithm, the design of calibration scheme and the design of the inversion algorithm. The research results have important practical significance for the research of using microwave radiometer to measure body temperature and subcutaneous tissue temperature.

Researchers have made a series of achievements in the field of non-contact measurement of human body temperature and subcutaneous tissue temperature by using total power microwave radiometer and Dicke microwave radiometer. In 2009, Bondst et al. [1,2] proposed to use Dicke microwave radiometer and printed dipole antenna for non-contact measurement of human body temperature. The measurement result showed that the Dicke microwave radiometer with printed dipole antenna can track the trend of physical temperature change of tissue model, but the measurement error was very large. In 2009–2012, Bondst et al. [2,3,4] improved the measurement antenna and designed an antenna probe with good directivity, which can radiate energy in uniaxial direction. In addition, they improved the system structure of the radiometer. The measurement results of the improved radiometer system were better than that of the Dicke microwave radiometer with printed dipole antenna proposed in Reference [1]. Around 2015, Li and Lang of Huazhong University of Science and Technology [5] conducted a study on the temperature measurement of heart ablation area in the cardiac radiofrequency ablation surgery with C-band Dicke microwave radiometer. Then they carried out contact and non-contact temperature measurement experiments on water. In 2017, Park and Jeong [6] produced a total power microwave radiometer for non-contact measurement of human body temperature. The radiometer and thermometer show good performance consistency in water temperature measurement from 298.15 to 316.25 K. The measurement error of radiometer is 0.90 K between 300.95–298.15 K by linear fitting method, the error reduced to 0.85 K by logarithmic fitting method.

In summary, total power microwave radiometer and Dicke microwave radiometer can measure temperature, but the temperature measurement error is large. The total power microwave radiometer cannot achieve ideal sensitivity due to various practical conditions, especially the adverse effect of gain variation on measurement accuracy. Dicke microwave radiometer has the disadvantages of complex structure and the errors caused by Dicke switch and reference load are difficult to overcome. In order to design a microwave radiometer with wider bandwidth, higher accuracy and higher sensitivity for non-contact measurement of human body temperature and subcutaneous tissue temperature, this paper proposes a design scheme of a novel interferometric microwave thermometer radiometer, which has higher theoretical sensitivity than total power microwave radiometer and Dicke microwave radiometer. In addition, the antenna with good directivity can improve detection accuracy. In this paper, a stepped fin line waveguide horn antenna with high directivity is used in the front end of microwave radiometer to reduce the influence of environment on the accuracy of temperature measurement.

The complex correlator is the core part of the microwave thermometer. In 2016, Kashi and Hu [7] developed an analog multiplicative complex correlator for passive millimeter wave imaging system. Due to the amplitude and phase imbalance of the power divider and combiner network in the whole frequency band, the radius of the correlation circle changed about 3.5 dB in 1 GHz bandwidth. In 2017, Wang et al. [8] developed an additive analog complex correlator with a bandwidth of 1 GHz. The test results showed that the complex correlator worked well under the broadband noise signal, the small correlation circle origin offset and root mean square error showed that the phase error of the correlator was very small, the change of correlation amplitude was only about 1 dB in 1 GHz, which was better than that of 3.5 dB in multiplication. In 2018, Wang et al. [9] developed a 3.5–8 GHz additive analog complex correlator for interferometric passive millimeter wave security imaging. The non-ideal characteristics of RF elements in this version of correlator caused quadrature amplitude error, which made the correlation circle closer to ellipse than to circle at some frequency points.

In conclusion, the analog multiplicative complex correlator has the disadvantage of limited detection bandwidth, the analog additive complex correlator has the disadvantages of complex structure, serious distortion under wide bandwidth and the detection sensitivity is limited by the square law detector. In this paper, an interferometric analog complex correlator is designed, which can not only simplify the structure of the complex correlator, but also make it work normally under the wide bandwidth.

The calibration of microwave radiometer can characterize the relationship between the output voltage of the radiometer and the brightness temperature received by the antenna [10]. In 2017, Park and Jeong [6] calibrated the radiometer with resistances of 22 and 50 Ω. Then, according to the reflection coefficient of the antenna, the insertion loss of the transmission line and the efficiency of the antenna, they deduced the brightness temperature received by antenna. In 2015, He [11] calibrated the radiometer with absorbing materials at room temperature and immersed in liquid nitrogen. However, due to the special storage conditions of liquid nitrogen, the measurement process was complex. When measuring the absorbing material in liquid nitrogen, the temperature of the antenna would be slightly higher than the actual physical temperature of the calibration source, therefore, it was necessary to integrate various errors to determine the antenna temperature of the absorbing material immersed in liquid nitrogen. In 2018, Dong et al. [12] designed a new grid calibration source which composed of polarization grid, artificial blackbody and cold air. The calibration source mixed the radiation energy of artificial blackbody and cold air through the rotation of polarization grid. Thus, the output of microwave radiation signal with controllable brightness temperature provided artificial temperature source for external calibration of microwave radiometer. In 2006, Li et al. [13] used a stepping motor to drive the radiometer to periodically receive radiation signals from two calibration loads.

In conclusion, calibration can be divided into step-by-step calibration and overall calibration. Step-by-step calibration calibrate the radiometer and antenna separately, and the overall calibration needs to select two calibration sources with different emissivity. In this paper, we design the step-by-step calibration scheme and the overall calibration scheme respectively. In step-by-step calibration, the radiometer is calibrated by microwave load, the antenna noise temperature is obtained by antenna efficiency and antenna physical temperature. Considering the complexity of calibration source production and calibration operation, the overall calibration scheme is designed by using the calibration sources with easily available and different emissivity.

The inversion algorithm establishes the relationship between the brightness temperature and the physical temperature of the target, then establishes the relationship between the output voltage of the radiometer and the physical temperature of the target. In 2013, Scheeler [14] designed a radiometer to measure human body temperature. The least square method and the optimal estimation method were used for temperature inversion and the advantages and disadvantages of the two inversion algorithms were demonstrated. In 2004, Gu [15] used BP neural network to inverse the apparent temperatures of cement ground, grassland, plastic runway and stealth coating targets according to the measured antenna temperature.

In conclusion, the least square method, the optimal estimation method and BP neural network algorithm can be used for temperature inversion. However, in the above inversion algorithms, they all assumed that the microwave radiometer only received the radiation energy of the target. The influence of temperature rise of microwave radiometer components on the output result was not considered. In this paper, the brightness temperature, aluminum plate temperature, antenna temperature, and feeder temperature are input into the inversion algorithm, the multiple linear regression algorithm, based on least square method, and BP neural network algorithm are used for temperature inversion.

## 2. Microwave Radiometer Temperature Measurement Scheme

In microwave radiation measurement, the microwave radiation power can be expressed by temperature [16], the simplified power-temperature relationship is shown in Equation (1):

(1) P=kT∆f

k is Boltzmann constant, T is absolute temperature, ∆f is bandwidth, and P is microwave radiation power. Thus, the temperature can be determined by measuring the output power of the microwave radiometer.

Microwave radiometer is key of non-contact rapid detection device for human body temperature. Microwave radiometer can be divided into total power microwave radiometer, Dicke microwave radiometer, and correlation radiometer, etc. according to circuit structure.

The detector of total power radiometer is usually a diode power detector, which completes power detection mainly using the unidirectional conductivity characteristics of the diode and the charging and discharging process of the detection load RC. The power series expansion of the current $i_{D}$ when the signal enters the diode is:

(2) $$i_{D}=a_{0}+a_{1}\left(V_{D}-V_{Q}\right)+a_{2}{\left(V_{D}-V_{Q}\right)}^{2}+a_{3}{\left(V_{D}-V_{Q}\right)}^{3}+\cdots$$

(3) $$i_{D}=a_{0}+a_{1}V_{i}+a_{2}V_{i}{}^{2}+a_{3}V_{i}{}^{3}+\cdots .$$

When the input is a sinusoidal signal $V_{i}t=A\cos\Omega t$, the current of the quadratic term is $a_{2}{\left(A\cos\Omega t\right)}^{2}$.

After the high frequency signal is filtered by the low pass filter, the average output voltage is:

(4) $$V_{\mathrm{AV}}=R\times [a_{0}+a_{2}{\left(A\cos\Omega t\right)}^{2}]=a_{0}R+a_{2}R(\frac{1}{2}A^{2}+\frac{1}{2}A^{2}\cos2\Omega t).$$

There are DC components $a_{0}R$ in the output voltage $V_{\mathrm{AV}}$, which cannot be filtered out by the low-pass filter. In addition, the temperature stability of the discrete diode circuit is poor, which will affect the detection accuracy of the total power radiometer.

**Figure 1:**
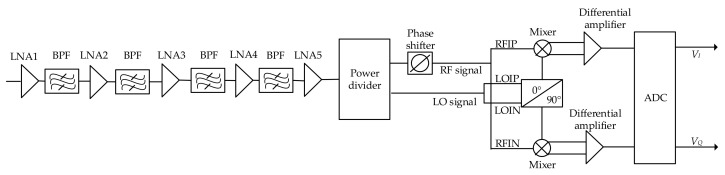
System block diagram of interferometric microwave radiometer.

An interferometric microwave radiometer is designed in this paper. The system block diagram of the interferometric microwave radiometer is shown in Figure 1.

The signal amplified and filtered by multi-stage low noise amplifier enters the microwave radiometer through the antenna, and then it is divided into two channel signals of equal amplitude and phase by the power divider. One of the radio frequency (RF) signals is mixing with local oscillator (LO) signal, the other RF signal is mixing with LO signal with 90° phase shift. After amplified by differential amplifier and sampled by ADC, the DC signal output from the correlation result represents the power value of the measured signal.

When the input signal is Acos (ωt), the output of the ideal radiometer is:

(5) $$V_{I}=\;\frac{A^{2}}{2}\cos\left(\varphi \right)\;$$

(6) $$\;V_{Q}=\;\frac{A^{2}}{2}\sin\left(\varphi \right)$$

$V_{I}$ is the *I*-channel output voltage of the ideal radiometer, $V_{Q}$ is the *Q*-channel output voltage of the ideal radiometer, A is the signal amplitude, φ is the system phase error caused by the circuit after the power divider. When the system phase error is small enough, the relationship between the input signal and the output signal is the square law.

Therefore, compared with total power microwave radiometer and Dicke microwave radiometer, the interferometric microwave radiometer has higher theoretical sensitivity. The amplitude and phase are shown in Equations (7) and (8):

(7) $$\sqrt{V_{I}{}^{2}+V_{Q}{}^{2}}=\sqrt{{\left(\;\frac{A^{2}}{2}\cos\left(\varphi \right)\right)}^{2}+{\left(\;\frac{A^{2}}{2}\sin\left(\varphi \right)\right)}^{2}}=\frac{A^{2}}{2}$$

(8) $$\arctan\left(\frac{V_{Q}}{V_{I}}\right)=\arctan\left(\frac{\frac{A^{2}}{2}\sin\left(\varphi \right)}{\frac{A^{2}}{2}\cos\left(\varphi \right)}\right)\;=\varphi$$

The output of the radiometer shows that the input power is proportional to the square of the voltage amplitude. Equation (1) has obtained the relationship between microwave radiation power and temperature, so the temperature can be determined by measuring the output voltage of the radiometer.

## 3. Design of Novel Interferometric Microwave Radiometer for Human Body Temperature Measurement

**Figure 2:**
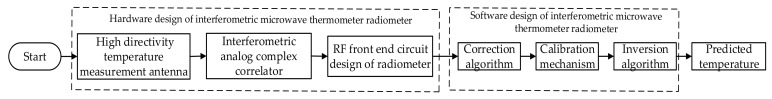
The design flow chart of the novel interferometric microwave thermometer radiometer.

The design flow chart of the novel interferometric microwave thermometer radiometer is shown in Figure 2.

The design of novel interferometric microwave thermometer radiometer includes hardware design and software design. The hardware design part includes the selection and simulation of high directivity temperature measurement antenna, the design of interferometric analog complex correlator and the design of RF front-end circuit. The software design part includes the design of correction algorithm, calibration scheme and inversion algorithm.

### 3.1. Hardware Design of Interferometric Microwave Thermometer Radiometer

#### 3.1.1. Design of High Directivity Temperature Measurement Antenna

When measuring body temperature with microwave radiometer, the aperture antenna is several times larger than wavelength, so a microwave radiometer working at high frequency can obtain good detection results without a large antenna aperture. However, the increase in frequency will reduce the detection depth, thereby reducing the incremental temperature $∆T_{E}$ produced by the subcutaneous hot spot. When frequency is 6 GHz, the microwave radiometer can detect the hot spots with depth of 5 cm and diameter of 2 cm [17]. The frequency band used in this design is 4–6 GHz.

**Figure 3:**
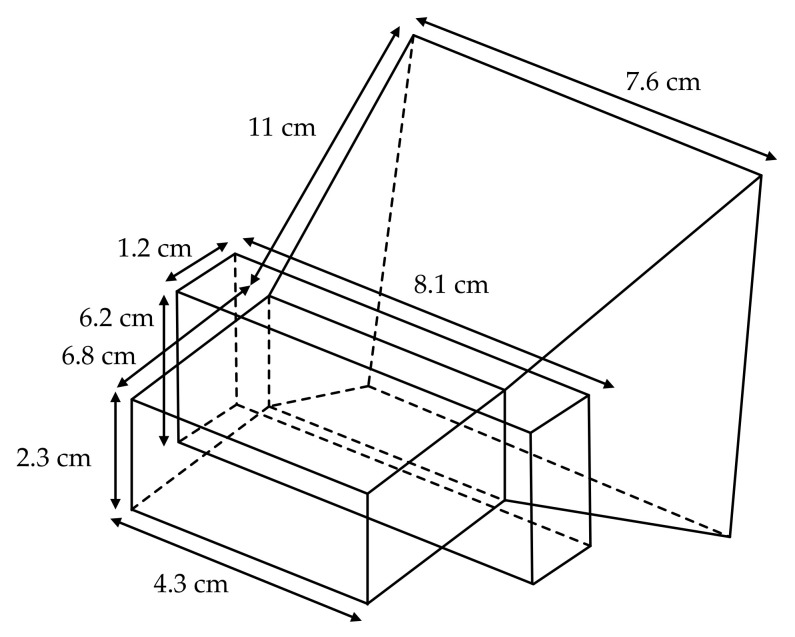
Dimension diagram of stepped fin line waveguide horn antenna.

In order to reduce the influence of environment on the accuracy of temperature measurement, a stepped fin line waveguide horn antenna with high directivity is used in the front end of microwave radiometer in this paper. The dimension diagram of the stepped fin line waveguide horn antenna is shown in Figure 3.

**Figure 4:**
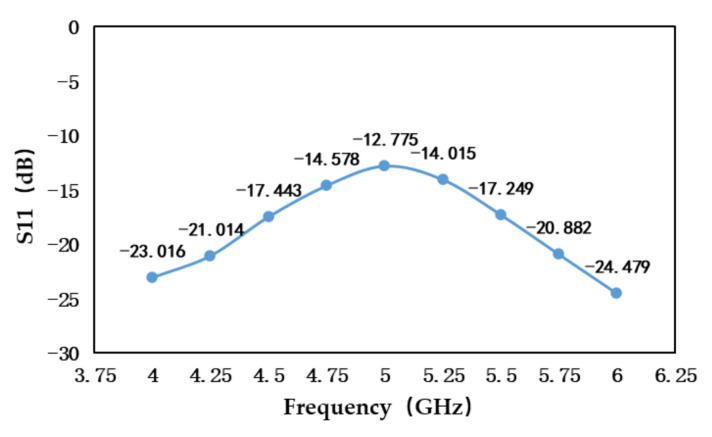
The reflection coefficient of the antenna.

Due to the distance between the antenna and the measured area is about 10 cm when the microwave radiometer measures the target temperature, it is necessary to match the aperture size with the measured area to reduce the influence of temperature in non-target areas on measurement results [18]. In addition, the side lobe and back lobe of the antenna can also receive the temperature of the surrounding environment. In order to ensure that the antenna receives as little radiation energy as possible from non-target objects, the temperature measurement antenna used in this paper is a high directivity waveguide horn antenna with good directional radiation characteristics, and the front and back rejection ratio is more than 20 dB. The reflection coefficient S11 of the antenna is shown in Figure 4.

**Figure 5:**
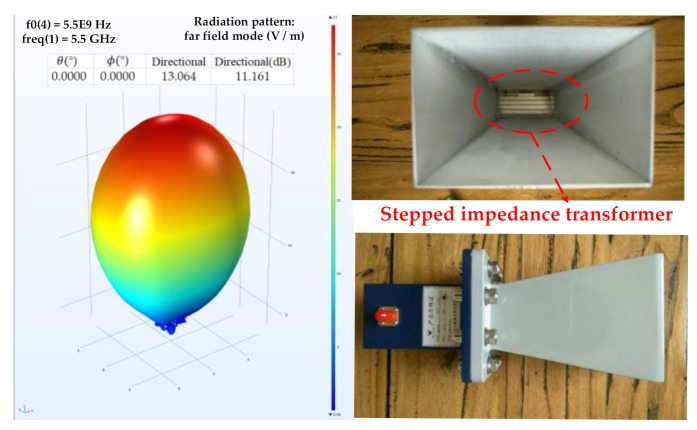
Radiation field distribution, directivity gain, stepped impedance transformer and antenna physical picture of horn antenna.

At the same time, the use of stepped impedance transformer can not only ensure that the antenna has a higher gain in the bandwidth above 2 GHz, but also the antenna used in this article has a smaller aperture area than the general horn antenna. The aperture of the antenna used in this paper is about 35$\;{\mathrm{cm}}^{2}$ and the antenna gain is about 12 dB. The radiation field distribution and directional gain of the antenna, stepped impedance transformer and antenna physical picture are shown in Figure 5.

#### 3.1.2. Design of Interferometric Analog Complex Correlator

The design and implementation of the interferometric microwave radiometer is an innovation in the field of microwave radiation measurement, and the complex correlator is the core part of the interferometric microwave radiometer. The electromagnetic wave radiated by non-target objects in the environment cause errors in the measured temperature value. The complex correlator can reduce the influence of non-target radiation on the measured temperature value and ensure the accuracy of the measured data by performing auto-correlation operation on the received signal. Therefore, the design of high-performance complex correlator is of great significance for microwave radiation measurement. The complex correlator is divided into digital complex correlator and analog complex correlator. The analog complex correlator is more suitable for the application of body temperature measurement because of its wide bandwidth, high sensitivity, and high processing speed [19].

Analog complex correlator can be divided into additive analog complex correlator and multiplicative analog complex correlator [20]. The additive analog complex correlator has the advantages of low cost and wide bandwidth, but the structure is complex, the distortion is serious under wide bandwidth [21] and the detection sensitivity is limited by the detection sensitivity of diode square law detector. At present, the maximum detection sensitivity of diode square law detector on the market is generally less than 55 mV/dBm, for example, ADL5906 module from ADI. Multiplicative analog complex correlator whose structure is simple uses multiplier to carry out complex correlation operation on received signal. However, the bandwidth is limited by the existing multiplier chip, so it is not suitable for wideband signal detection. In addition, there is large phase error in the input channel, which will affect the temperature measurement accuracy.

In order to design an analog complex correlator which can be used in the field of temperature measurement, this paper innovatively proposes a design scheme of interferometric complex correlator. The interferometric complex correlator is mainly composed of power divider, phase shifter, quadrature demodulator, low-pass filter, and fully differential amplifier. Compared with the traditional analog complex correlator, it has the advantages of wider bandwidth, simpler circuit structure and higher detection sensitivity.

In this paper, a wideband quadrature demodulator is used to design complex correlator. First, the signal is connected to the power divider and divided into two equal half power signals. Second, a programmable phase shifter which can correct the phase error by controlling the phase-shift value according to the phase value of two-channel is added to one of the input signals. Finally, the two pre-processed signals are respectively connected to the RF input and LO input of the quadrature demodulator, the LO signal is mixed with RF signal after phase shift of 0° and 90° respectively to complete the autocorrelation operation of input signal. The autocorrelation operation can reduce the influence of non-target radiation on temperature measurement accuracy. In this design, 500–6000 MHz wideband quadrature demodulator is selected to detect wideband signal and simplify the circuit structure.

**Figure 6:**
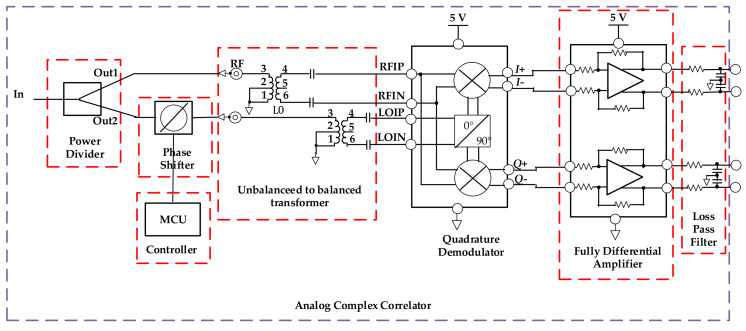
Design block diagram of complex correlator.

The output signal after autocorrelation operation is not only small, but also contains common-mode noise. Therefore, a fully differential amplifier is used to amplify the output signal, and a low-pass filter is used to retain the voltage signal which representing human body temperature, then the voltage signal is uploaded to the PC for analysis and correction. Figure 6 shows the block diagram of the complex correlator.

**Figure 7:**
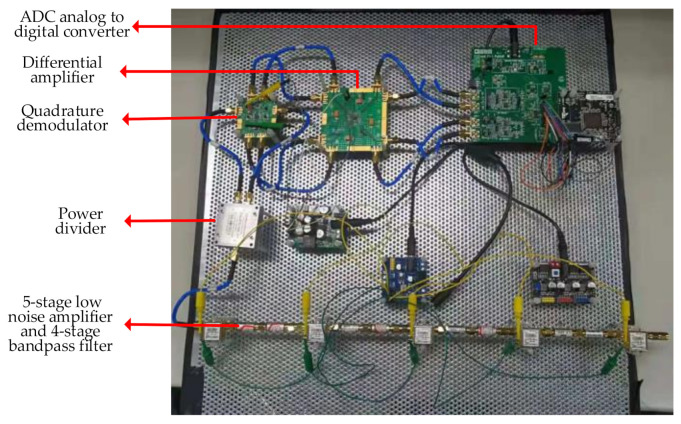
Prototype of interferometric microwave thermometry radiometer.

Figure 7 shows the prototype of interferometric microwave thermometry radiometer. In order to calculate the detection sensitivity of the complex correlator, the microwave radiometer receives the electromagnetic wave of signal source, the quadrature demodulator performs the autocorrelation operation, and the ADC samples the voltage data and uploads it to PC. If the output voltage amplitude of channel *I* and channel *Q* are equal and the phase difference is 90°, the performance of the complex correlator is good, otherwise software correction algorithm is required [8].

**Figure 8:**
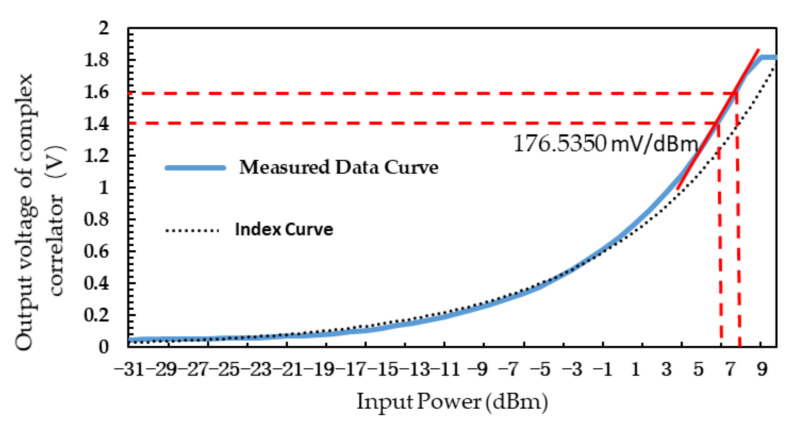
Relationship between input power and output voltage of interferometric complex correlator.

Figure 8 shows the relationship between input power and output voltage of interferometric complex correlator when the frequency of the signal source is 5 GHz.

It can be seen from Figure 8 that there is an exponential relationship between the input power and the output voltage of the interferometric complex correlator. When the input power is set at 7 dBm, the output voltage changes the most. At this time, the complex correlator has the highest detection sensitivity of 176.54 mV/dBm, the temperature measurement accuracy is the highest, and 1 mV voltage change corresponds to about 0.0057 dBm change in input power. The human body temperature ranges from 35 to 45 °C. From Equations (9)–(11):

(9) $$KT=10\times \log(1.38\times 10^{-23}\times T)+30$$

(10) BW=10log(BandWidth)

(11) Pwr1=KT+BW

K is Boltzmann constant, T is absolute temperature, BW is bandwidth, Pwr1 is noise power. The power difference in this range is shown in Equation (12):

(12) $$\Delta \mathrm{Pwr}=10\times \log(1.38\times 10^{-23}\times 318.15K)+30+10\log(\mathrm{BandWidth})\;-10\times \log(1.38\times 10^{-23}\times 308.15K)+30\;-10\log(\mathrm{BandWidth})\approx 0.14\;\mathrm{dBm}.$$

Similarly, a power difference of 0.0014 dBm can be obtained when the temperature changes by 0.1 °C. Then 1 mV corresponds to:

(13) $$\frac{0.0057}{0.0014}\times 0.1\approx 0.40{\;}^{o}C/\mathrm{mV}.$$

That is, the temperature sensitivity is about $0.40{\;}^{o}C/\mathrm{mV}$. The temperature sensitivity can be further improved after the microwave radiometer is corrected by the software algorithm.

Compared with the traditional diode square law detector, the analog complex correlator designed in this paper has many advantages. When the input power is −9 to −10 dBm, the detection sensitivity of diode square law detector is 9.2 mV/dBm [21], while the detection sensitivity of the analog complex correlator designed in this paper is 32 mV/dBm. The detection sensitivity increases by 3.48 times and the maximum sensitivity can reach 176.54 mV/dBm. The traditional diode square law detection requires that the power of the input signal to be small, but the error caused by small signal is large, at the same time, the principle of traditional diode square law detection is based on the small signal approximation model, which has nonlinear distortion. The analog complex correlator designed in this paper overcomes the shortcoming of requiring the input signal to be small signal and the good multiplication performance ensures the detection accuracy without distortion.

#### 3.1.3. RF Front End Circuit Design of Microwave Radiometer

According to the Equations (9)–(11), when the bandwidth is 2 GHz, the noise power is about −80.70 to −80.56 dBm in the temperature range of 35–45 °C. Considering the loss of other modules, adapter, coaxial line and the gain of antenna, the total gain of multi-stage low noise amplifier used in RF front-end should be about 90 dBm, so that the complex correlator can reach the working point around 7 dBm of maximum sensitivity, furthermore, it ensures the microwave radiometer can obtain higher detection sensitivity [22].

**Figure 9:**
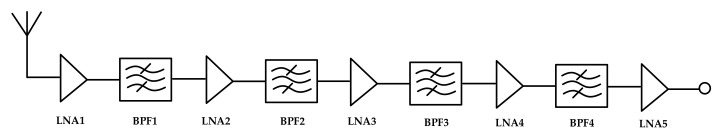
Block diagram of radio frequency (RF) front end of microwave radiometer (including 5-stage low noise amplifier and 4-stage bandpass filter).

Figure 9 shows the block diagram of RF front-end of microwave radiometer, which is composed of 5-stage low-noise amplifier and 4-stage band-pass filter. In this design, three low noise amplifiers (ZX60-83LN-S+, Mini-Circuits, Shanghai, China) with noise figure of 1.59 dB and gain of 20 dB are placed in the front of RF front-end, which can effectively reduce the noise figure of microwave radiometer system. And it is cascaded with two 15 dB low noise amplifiers (ZX60-V63+, Mini-Circuits, Shanghai, China) to make the RF front-end gain reach 90 dB. At the same time, a group of band-pass filters are added between every two stages of low noise amplifier to limit the bandwidth of microwave radiometer. Each group of band-pass filters consists of a high pass filter (VHF-3800+, Mini-Circuits, Shanghai, China) and a low-pass filter (VLF-5500+, Mini-Circuits, Shanghai, China).

**Figure 10:**
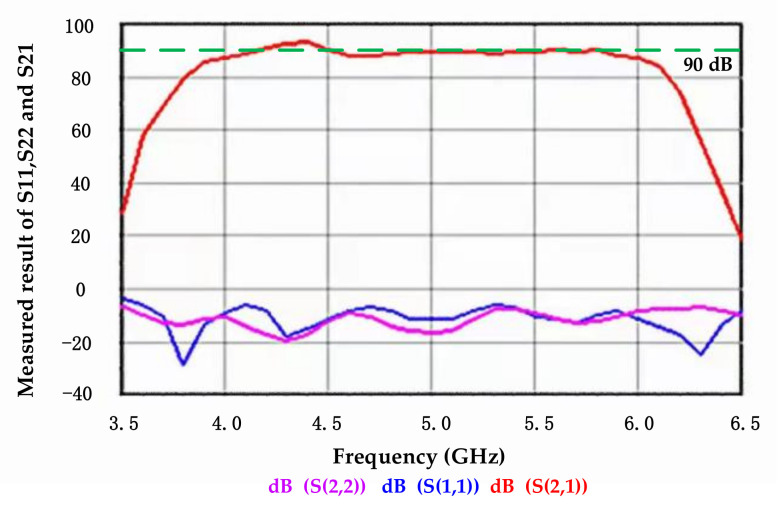
Measured result of S11, S22, and S21 of RF front end circuit.

Figure 10 shows the measured result of S11, S22, and S21 of the RF front end. S11 is the input reflection coefficient, which namely the input return loss. S22 is the output reflection coefficient, which namely the output return loss. S21 is the forward transmission coefficient, which namely the gain. The frequency range of the microwave radiometer is from 4 to 6 GHz. The gain in the working bandwidth is 88.5 ± 1.5 dB. The return loss of input and output is better than −7 dB.

### 3.2. Collection and Processing of Water Temperature Data

It is convenient to use a model with electromagnetic characteristics similar to human tissue for temperature measurement. Debye model of tissue dielectric constant showed that muscle, blood, and skin (wet) have higher moisture content. Vaks et al. [23] measured and inversed the water whose physical temperature is linearly distributed with depth in 1 GHz frequency band. Popovic et al. [24] measured and inversed the water in each layer whose physical temperature varies with depth in the frequency band of 1.4 and 2.7 GHz. They both achieved correct result. Therefore, this paper also uses water as the measurement object to study the performance of the interferometric microwave radiometer.

**Figure 11:**
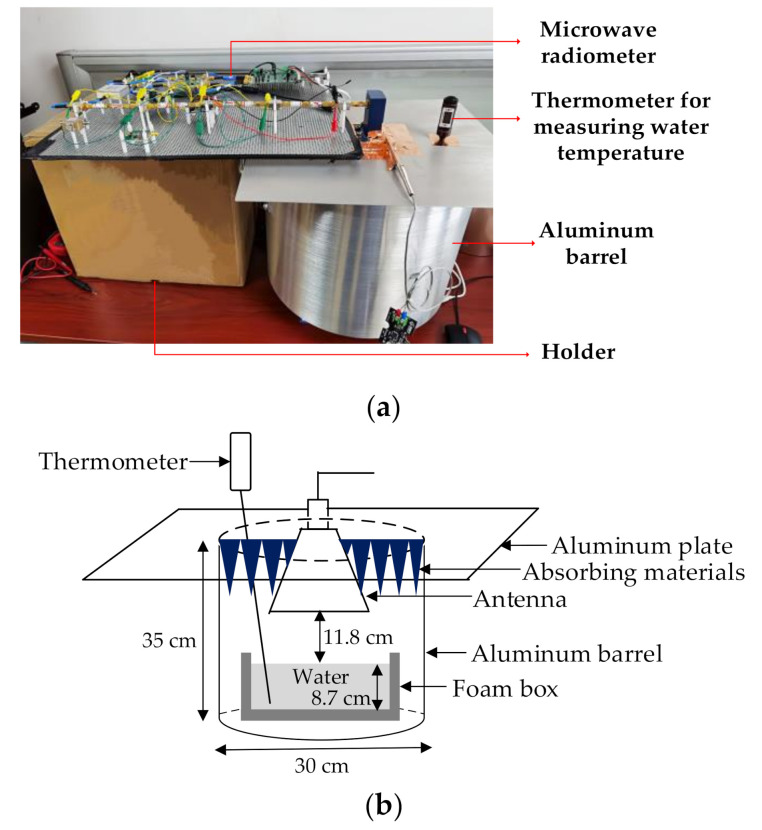
Device diagram of novel interferometric microwave radiometer. (**a**) Actual picture of interferometric microwave radiometer; (**b**) diagram of signal acquisition device of interferometric microwave radiometer).

Figure 11 shows the device diagram of the interferometric microwave thermometer. The rectangular waveguide of the antenna is sealed in the aluminum barrel, which contains a foam boxes filled with water, the antenna is facing the water for measurement. The antenna aperture is surrounded by absorbing materials in order to reduce the influence of electromagnetic interference. The aluminum barrel can also reduce the influence of electromagnetic interference from the external environment. A water temperature meter with an accuracy of ±0.1 °C/°F is inserted into the aluminum barrel to measure the actual water temperature for comparison with the predicted temperature.

Due to the high sensitivity of the microwave radiometer, the external electromagnetic radiation will affect the output of the microwave radiometer. In the actual test process, the absorbing material is placed outside the microwave radiometer to reduce the influence of electromagnetic wave.

**Figure 12:**
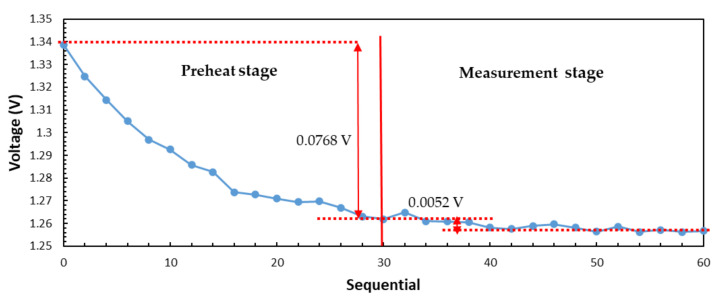
Actual water temperature test result of interferometric microwave radiometer.

Figure 12 shows the water temperature test result. When the microwave radiometer is in the preheat stage, the initial measured voltage is high and the voltage change is 0.0768 V. With the gradual stability of circuit components, the measured voltage value which change is only 0.0052 V also tends to be stable. The time of preheat stage is about 30 min.

The microwave radiometer is used to measure the water sealed in aluminum barrel with temperature range of 33–55 °C, and the performance of the microwave radiometer can be evaluated by observing the change in output voltage when the temperature changes. In the experiment, 2048 samples are collected from each temperature point and the mean voltage and mean phase are calculated. Table 1 shows the mean output voltage of the microwave radiometer when measuring 33–55 °C water sealed in aluminum barrel.

According to the water temperature test result in Table 1, the change of output voltage of the microwave radiometer can be obtained when the temperature changes by 0.1 °C. The average voltage change is 17.755 mV/°C, which is the result of the non-contact water temperature measurement of the interferometric microwave radiometer.

**Figure 13:**
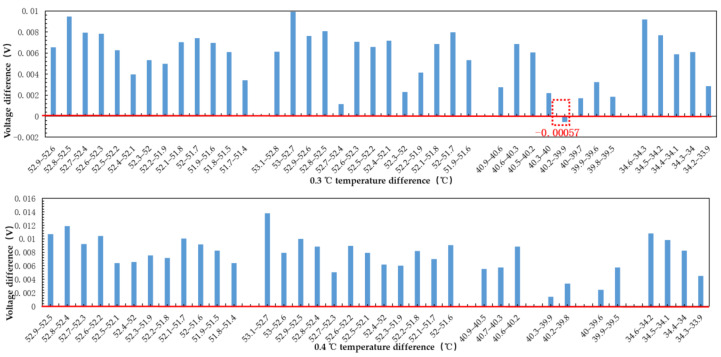
Mean voltage value of water at 33–55 °C measured by microwave radiometer.

As can be seen from the result in Figure 13, when the temperature changes by 0.3 °C, there will be the situations that water with high temperature and low output voltage, so the radiometer cannot distinguish the temperature changes of 0.3 °C. When the temperature changes by 0.4 °C, all test data meet the requirement that the voltage of water with high temperature is high. That is, the microwave radiometer can distinguish the temperature changes of 0.4 °C. In other words, the temperature resolution of the microwave radiometer designed in this paper reaches 0.4 K.

**Figure 14:**
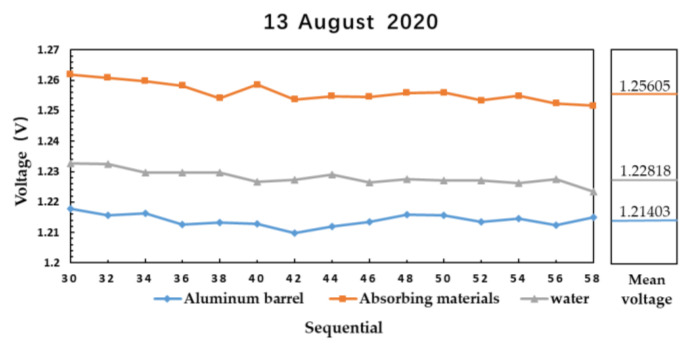
The result of microwave radiometer measuring aluminum plate, absorbing material, and water sealed in aluminum barrel on 13 August 2020.

**Figure 15:**
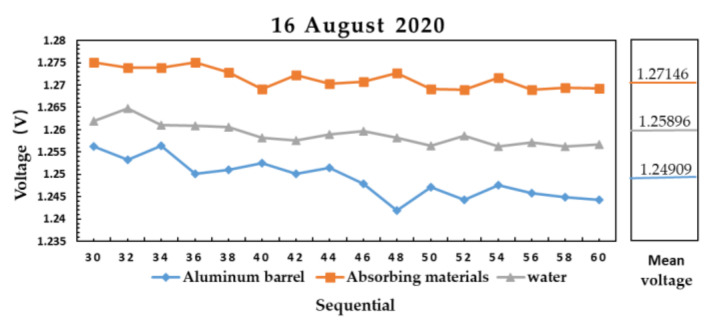
The result of microwave radiometer measuring aluminum plate, absorbing material, and water sealed in aluminum barrel on 16 August 2020.

The results of microwave radiometer measuring aluminum plate, absorbing material and water are shown in Figure 14 and Figure 15.

Figure 14 shows the measured data of aluminum plate at 27 °C, absorbing material at 28.4 °C and water at 28 °C on 13 August 2020. Figure 15 shows the measured data of aluminum plate at 26.5 °C, absorbing material at 27.8 °C and water at 28 °C on 16 August 2020. The output voltage of microwave radiometer on the same day is $V_{\mathrm{absorbing}\;\mathrm{material}}>V_{\mathrm{water}}>V_{\mathrm{aluminum}\;\mathrm{plate}}$, and the difference of the average voltage between absorbing material and water is larger than that between water and aluminum plate.

The relationship between brightness temperature $T_{B}$ and physical temperature *T* is:

(14) $$T_{B}=T\times \varepsilon$$

ε is emissivity. In the 4–6 GHz microwave band, the emissivity of aluminum plate is estimated to be about 0.26 [17], and the emissivity of absorbing material is estimated to be about 0.995 [17]. The relationship between brightness temperature and output voltage is linear, in Figure 14:

(15) $$\frac{V_{\mathrm{absorbing}\;\mathrm{materials}}-V_{\mathrm{water}}}{V_{\mathrm{water}}-V_{\mathrm{aluminum}\;\mathrm{plate}}}=\frac{T_{\mathrm{absorbing}\;\mathrm{materials}}\times \varepsilon_{\mathrm{absorbing}\;\mathrm{materials}}-T_{\mathrm{water}}\times \varepsilon_{\mathrm{water}}}{T_{\mathrm{water}}\times \varepsilon_{\mathrm{water}}-T_{\mathrm{aluminum}\;\mathrm{plate}}\times \varepsilon_{\mathrm{aluminum}\;\mathrm{plate}}}\;\frac{1.256-1.228}{1.228-1.214}=\frac{28.4\times 0.995-28\times \varepsilon_{\mathrm{water}}}{28\times \varepsilon_{\mathrm{water}}-27\times 0.26}\;\varepsilon_{\mathrm{water}}\approx 0.53$$

(16) $$\begin{array}{c} \mathrm{Detection}\;\mathrm{sensitivity}\\ =\frac{T_{\mathrm{absorbing}\;\mathrm{materials}}\times \varepsilon_{\mathrm{absorbing}\;\mathrm{materials}}-T_{\mathrm{aluminum}\;\mathrm{plate}}\times \varepsilon_{\mathrm{aluminum}\;\mathrm{plate}}}{V_{\mathrm{absorbing}\;\mathrm{materials}}-V_{\mathrm{aluminum}\;\mathrm{plate}}}\\ =\frac{21.238}{0.042}\approx 0.51\;℃/\mathrm{mV} \end{array}$$

In Figure 15:

(17) $$\frac{V_{\mathrm{absorbing}\;\mathrm{materials}}-V_{\mathrm{water}}}{V_{\mathrm{water}}-V_{\mathrm{aluminum}\;\mathrm{plate}}}=\frac{T_{\mathrm{absorbing}\;\mathrm{materials}}\times \varepsilon_{\mathrm{absorbing}\;\mathrm{materials}}-T_{\mathrm{water}}\times \varepsilon_{\mathrm{water}}}{T_{\mathrm{water}}\times \varepsilon_{\mathrm{water}}-T_{\mathrm{aluminum}\;\mathrm{plate}}\times \varepsilon_{\mathrm{aluminum}\;\mathrm{plate}}}\;\frac{1.271-1.259}{1.259-1.249}=\frac{27.8\times 0.995-28\times \varepsilon_{\mathrm{water}}}{28\times \varepsilon_{\mathrm{water}}-26.5\times 0.26}\;\varepsilon_{\mathrm{water}}\approx 0.39$$

(18) $$\begin{array}{c} \mathrm{Detection}\;\mathrm{sensitivity}\\ =\;\frac{T_{\mathrm{absorbing}\;\mathrm{materials}}\times \varepsilon_{\mathrm{absorbing}\;\mathrm{materials}}-T_{\mathrm{aluminum}\;\mathrm{plate}}\times \varepsilon_{\mathrm{aluminum}\;\mathrm{plate}}}{V_{\mathrm{absorbing}\;\mathrm{materials}}-V_{\mathrm{aluminum}\;\mathrm{plate}}}\\ =\frac{20.771}{0.022}\approx \;0.93\;℃/\mathrm{mV}. \end{array}$$

In the 4–6 GHz microwave band, the estimated emissivity of water is between 0.35–0.6 [17], which is consistent with the calculation result. It is proved that the microwave radiometer has the ability to distinguish materials with different emissivity.

**Figure 16:**
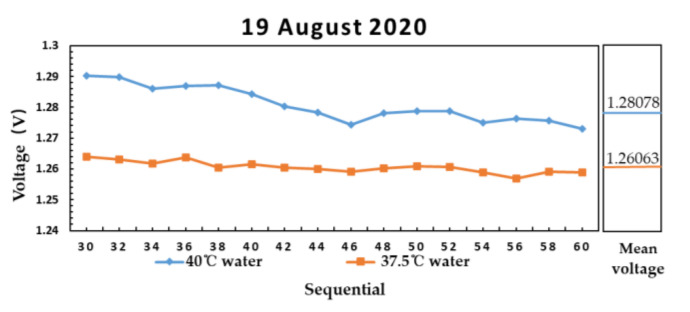
The measurement result of water at 40 and 37.5 °C on 19 August 2020.

Figure 16 shows the measurement result of water at 40 and 37.5 °C on 19 August 2020.

**Figure 17:**
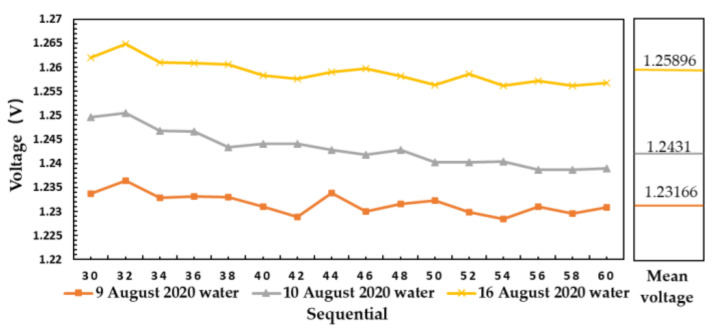
Result of microwave radiometer measuring water at different temperature in different days.

As shown in Figure 17, the water temperature of 27.5 °C was measured on 9 August 2020, 27.6 °C water was measured on 10 August 2020, 28 °C water was measured on 16 August 2020, respectively.

According to Equation (14), when the emissivity is constant, the brightness temperature of the material with high physical temperature is also high. The results in Figure 16 and Figure 17 are consistent with the theoretical brightness temperature. It is proved that the microwave radiometer can distinguish water with different temperature on the same day and on different days.

In Figure 17, according to the relationship between brightness temperature and output voltage:

(19) $$\frac{V_{\mathrm{absorbing}\;\mathrm{materials}}-V_{\mathrm{water}}}{V_{\mathrm{water}}-V_{\mathrm{aluminum}\;\mathrm{plate}}}=\frac{T_{\mathrm{absorbing}\;\mathrm{materials}}\times \varepsilon_{\mathrm{absorbing}\;\mathrm{materials}}-T_{\mathrm{water}}\times \varepsilon_{\mathrm{water}}}{T_{\mathrm{water}}\times \varepsilon_{\mathrm{water}}-T_{\mathrm{aluminum}\;\mathrm{plate}}\times \varepsilon_{\mathrm{aluminum}\;\mathrm{plate}}}\;\frac{1.259-1.243}{1.243-1.232}=\frac{28\times \varepsilon_{\mathrm{water}}-27.6\times \varepsilon_{\mathrm{water}}}{27.6\times \varepsilon_{\mathrm{water}}-27.5\times \varepsilon_{\mathrm{water}}}\;{\;\varepsilon }_{\mathrm{water}}\approx 0.35.$$

The test result of water in different days and different temperatures are in accordance with the law of water emissivity, which proves that the measuring device has good shielding effect for electromagnetic interference.

**Figure 18:**
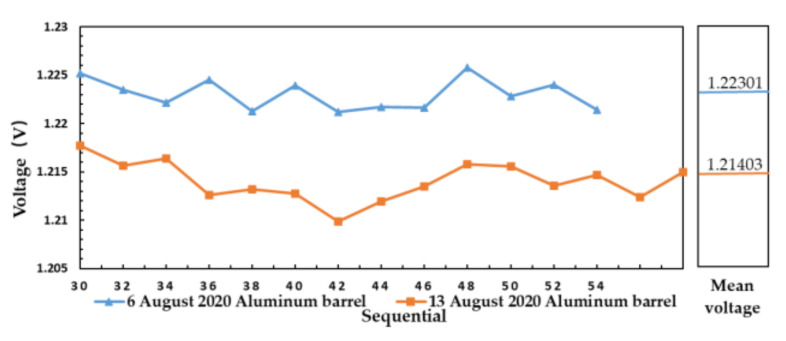
The measurement result of aluminum plate with different temperature in different days.

Figure 18 shows the measurement result of aluminum plate at 28.8 °C on 6 August 2020 and aluminum plate at 27 °C on 13 August 2020.

According to Equation (14), the brightness temperature of aluminum plate is shown in Table 2:

The output voltage value of high brightness temperature value is also high, which proves that the microwave radiometer can distinguish aluminum plates with different temperatures in different days, it also shows the applicability of the microwave radiometer for a variety of measured objects. The measurement results of different days meet the consistency of brightness temperature and output voltage, which indicates that electromagnetic interference has little influence on the output result.

Table 3 shows the comparison of temperature resolution of different types of microwave radiometers.

It can be seen from Table 3 that the temperature resolution of the interferometric microwave radiometer designed in this paper is higher than that of the total power microwave radiometer [6], the one-dimensional synthetic aperture microwave radiometer [25], the Ka-band direct detection radiometer [26], the W-band direct detection radiometer [27], the Dicke microwave radiometer without constant temperature treatment in reference [28] and Ka-band AC radiometer [29]. After designing the thermostat, the temperature resolution of the interferometric microwave radiometer designed in this paper will be further improved. The temperature resolution of Dicke microwave radiometer in reference [5] was measured according to the resistance at different temperatures, while the 0.4 K temperature resolution in this paper was obtained by using the whole system to measure water. The non-contact water temperature measurement result of the Dicke microwave radiometer designed in reference [5] is 7.36 mV/°C and that of the interferometric microwave radiometer designed in this paper is 17.755 mV/°C. That is, the temperature measurement result of the interferometric microwave radiometer designed in this paper is better than that of the Dicke microwave radiometer in Reference [5].

This paper innovatively designs the hardware structure of the interferometer microwave radiometer. The water temperature measurement results show that the temperature resolution of the interferometer microwave radiometer designed in this paper is better than that of the total power microwave radiometer [6], the Dicke microwave radiometer [5,28], the one-dimensional synthetic aperture microwave radiometer [25], the Ka-band direct detection radiometer [26], the W-band direct detection radiometer [27] and Ka-band AC radiometer [29]. Through the design of correction algorithm, calibration scheme and inversion algorithm, the temperature measurement accuracy of the microwave radiometer will be further improved. The results of this project are of great significance to the research of accurate, rapid, and non-contact human body temperature measurement.

### 3.3. Design of Temperature Measurement Software for Microwave Radiometer

#### 3.3.1. Microwave Radiometer Offset Error Correction Algorithm 

When the input of the *I* and *Q* channels are two signals with the same frequency and the system phase error is ∅, the theoretical output voltage values of *I* and *Q* channels of the complex correlator are shown in Equations (7) and (8). The phase error, gain error, and offset error of the microwave radiometer can affect the temperature measurement accuracy. In order to ensure the temperature measurement accuracy of the microwave radiometer, the error should be corrected.

First, the microwave radiometer is connected to a sine signal with the frequency is 5 GHz and the power is 0 dBm. Second, the main controller STM32 (Shenzhen Youxin Electronic Technology Co., Ltd., Shenzhen, China) controls the phase shifter to sweep the phase by 90° step. Then, 2048 groups of *I*/*Q* channel voltage values are collected at each phase point. Finally, the results are stored in the specified path. according to:

(20) $$V_{I}\;=\;10\;\times \;I/65535\;-\;5$$

(21) $$V_{Q}\;=\;10\;\times \;Q/65535\;-\;5$$

(22) $$\emptyset =\frac{\sum_{i=1}^{2048}\arctan\left(\frac{V_{Qi}}{V_{Ii}}\right)}{2048}.$$

Calculating the voltage *V* and phase ∅ at each phase point. The phase error is the difference between the theoretical phase value and ∅, the theoretical phase difference between *I*/*Q* channel is 90°.

The voltage and phase mean values of some sampled data are shown in Table 4.

It can be seen from Table 4 that the actual phase difference between channel *I* and channel *Q* is about 50°, which means that the phase error between channel *I* and channel *Q* is about 40°.

The phase error of the system is obtained by averaging the phase error of the sampled data, and then the phase shift value of the phase shifter is controlled to compensate the system phase error. After phase compensation, the relationship between the voltage and the sampled value should be linear, the intercept is offset error $e_{\mathrm{offset}}$, and the slope *S* is the parameter of gain error $e_{\mathrm{gain}}$. The ideal offset error $e_{\mathrm{offset}\_\mathrm{ideal}\;}$ and ideal slope $S_{\mathrm{ideal}\;}$ are:

(23) $$e_{\mathrm{offset}\_\mathrm{ideal}\;}=\;0$$

(24) $$S_{\mathrm{ideal}\;}=\frac{Code_{\max\;}-Code_{\min\;}}{+V_{\mathrm{REF}}-\left(-V_{\mathrm{REF}}\right)}$$

The correction equation for offset error $e_{\mathrm{offset}\_\mathrm{correction}}$ and gain error $e_{\mathrm{gain}\_\mathrm{correction}}$ is:

(25) $$e_{\mathrm{offset}\_\mathrm{correction}}=-e_{\mathrm{offset}}$$

(26) $$e_{\mathrm{gain}\_\mathrm{correction}\;}=\frac{S_{\mathrm{ideal}\;}}{S_{\mathrm{actual}\;}}$$

$S_{\mathrm{actual}\;}$ is actual slope. Thus:

(27) $$Code_{\mathrm{correction}\;}=\frac{Code_{\mathrm{receive}\;}\times S_{\mathrm{ideal}\;}}{S_{\mathrm{actual}\;}}+e_{\mathrm{offset}\_\mathrm{correction}}$$

$Code_{\mathrm{correction}\;}$ is corrected sample value. $Code_{\mathrm{receive}\;}$ is received sample values.

In the experiment, the intercept and slope parameters are obtained by linear fitting method, and then the gain error and offset error are corrected according to error correction method.

**Figure 19:**
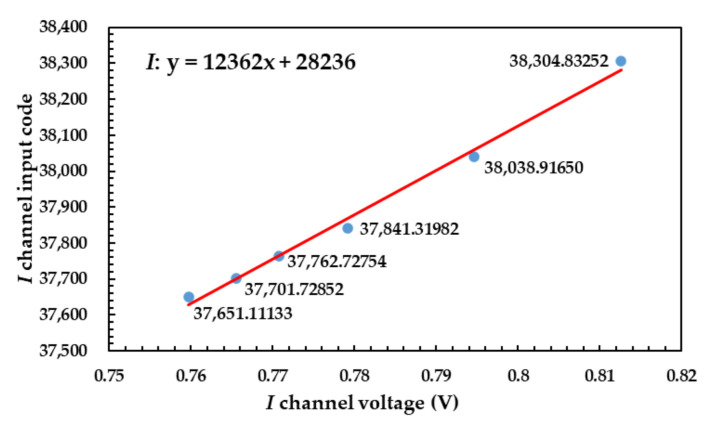
Corrected result of *I* channel using correction algorithm.

The corrected result of *I* channel is shown in Figure 19.

**Figure 20:**
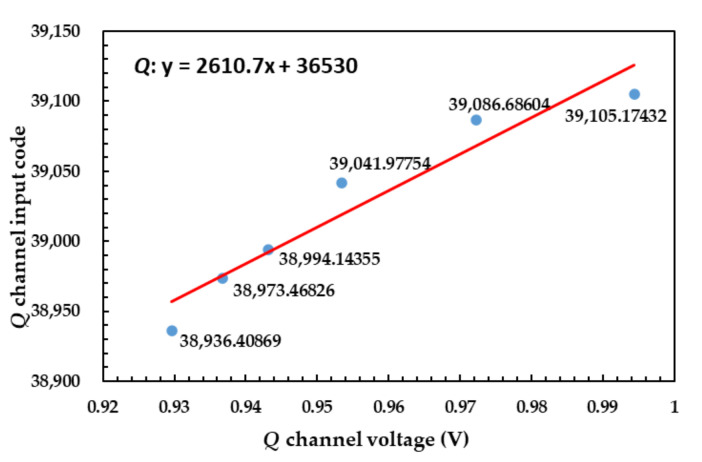
Corrected result of *Q* channel using correction algorithm.

The corrected result of *Q* channel is shown in Figure 20.

The corrected results are shown in Table 5.

The result in Table 5 shows that the phase error reduces to about 1.4° after correction, and when the temperature changes 0.1 °C, the average value of voltage changes obviously. In other words, the correction algorithm can reduce the influence of phase error, offset error and gain error on the output result and improve the temperature measurement accuracy.

**Figure 21:**
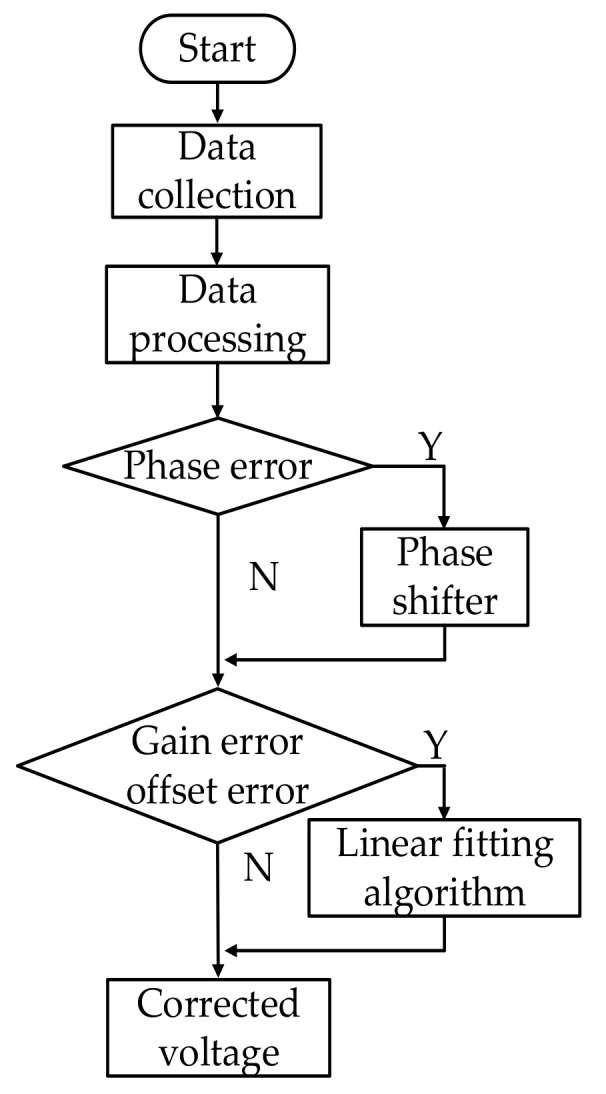
Software design flow chart of correction algorithm.

The software design flow chart of correction algorithm is shown in Figure 21.

#### 3.3.2. Design of Calibration Mechanism for Microwave Radiometer

The calibration of microwave radiometer is to construct the quantitative relationship between the output of the microwave radiometer and the received radiation value, by using the microwave radiometer to receive the radiation signal from the calibration source with accurate microwave radiation characteristics. Calibration algorithm is divided into step-by-step calibration and overall calibration [11].

**Figure 22:**
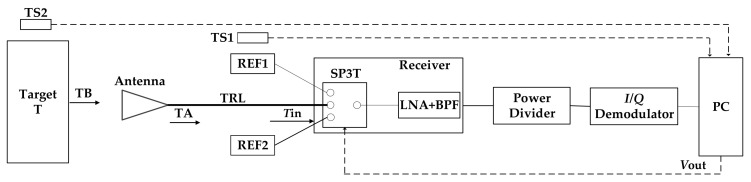
Schematic diagram of interferometric microwave radiometer with double reference load.

Step-by-step calibration is to calibrate the radiometer and antenna respectively. The radiometer is calibrated with 50 and 75 Ω microwave loads in this paper. The circuit structure is shown in Figure 22.

When 50 Ω microwave load is connected, the output of microwave radiometer $V_{\mathrm{out}2}$ is 1.328 V. When 75 Ω microwave load is connected, the output of microwave radiometer $V_{\mathrm{out}1}$ is 1.204 V. As the reference characteristic impedance of microwave load is $Z_{0}=50\;\Omega$ and the physical temperature $T_{r}$ is 30.3 °C, the equivalent noise temperature $T_{REF}$ is [11]:

(28) $$T_{\mathrm{REF}}=\left(1-{\left|\Gamma_{\mathrm{REF}}\right|}^{2}\right)T_{r}.$$

Among:

(29) $$\Gamma_{\mathrm{REF}}=\frac{Z_{0}-R_{\mathrm{REF}}}{Z_{0}+R_{\mathrm{REF}}}.$$

The temperature $T_{\mathrm{REF}2}$ corresponding to 50 Ω is 303.45 K and the temperature $T_{\mathrm{REF}1}$ corresponding to 75 Ω is 291.312 K. Then the system gain $G_{T}$ is:

(30) $$G_{T}=\frac{V_{\mathrm{out}2}-V_{\mathrm{out}1}}{T_{\mathrm{REF}2}-T_{\mathrm{REF}1}}=\frac{1.328-1.204}{303.45-291.312}=0.01.$$

**Figure 23:**
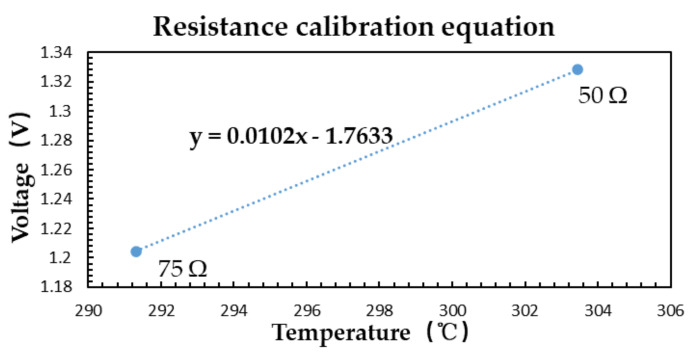
Calibration curve of microwave load.

The calibration curve of microwave load is shown in Figure 23:

According to the calibration curve, the system noise voltage of radiometer is 1.023 V. The sampling results of absorbing materials at 28.3 °C are used as verification data, as shown in Table 6.

According to the Equations (31)–(33):

(31) $$V_{\mathrm{out},A}-V_{\mathrm{outi}}=G_{T}\left(T_{\mathrm{ini}}-T_{\mathrm{REFi}}\right)$$

(32) $$T_{\mathrm{ini}}=T_{\mathrm{REFi}}+\frac{V_{\mathrm{out},A}-V_{\mathrm{outi}}}{G_{T}}$$

(33) $$T_{\mathrm{in}}=\frac{1}{2}\left(T_{\mathrm{in}1}+T_{\mathrm{in}2}\right)$$

$V_{\mathrm{out},A}$ is corrected voltage of object. The brightness temperature $T_{\mathrm{in}}$ entering the radiometer is:

(34) $$T_{\mathrm{in}}\;=\;295.828\;K\;=\;22.678\;{}^{\circ}C.$$

The brightness temperature of the absorbing material measured by the antenna is:

(35) $$T_{A}=\varepsilon_{\mathrm{ab}}\times T_{\mathrm{ab}}$$

$T_{A}$ is brightness temperature of absorbing material,${\;\varepsilon }_{\mathrm{ab}}$ is emissivity of absorbing material, and $T_{\mathrm{ab}}$ is physical temperature of absorbing material.

The antenna is lossy and it absorbs part of the microwave noise power. The antenna radiation efficiency η is usually used to describe the antenna loss. The antenna also has self-radiation (1 − η)$T_{0}$, $T_{0}$ is the physical temperature of the antenna. The noise temperature $T_{a}$ at the output end of the lossy antenna is equal to the noise temperature $\eta T_{A}$ transmitted through the antenna plus the self-radiation contribution of the antenna itself [17]. As is shown in Equation (36):

(36) $$T_{a}=\eta T_{A}+(1-\eta )T_{0}.$$

The radiation efficiency of the antenna is 70% by simulation, and the noise temperature $T_{a}$ of the antenna output is calculated by the measured data of absorbing materials:

(37) $$T_{a}=\;0.7\;\times \;28.1585\;+\;0.3\;\times \;27.3\;=\;27.9010.$$

The loss of the antenna is:

28.1585 − 27.9010 = 0.2575 °C.(38)

Therefore, the brightness temperature of absorbing material at 28.4 °C obtained by the step-by-step calibration scheme is:

22.678 + 0.2575 ≈ 22.94 °C.(39)

There are many intermediate parameters in the step-by-step calibration and the process of antenna calibration is complex, so the accuracy of step-by-step calibration is low.

**Figure 24:**
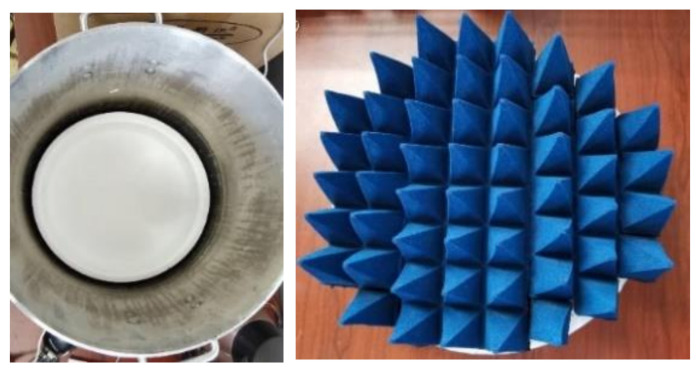
The (**left**) figure shows the calibration source of aluminum plate, the (**right**) figure shows the calibration source of absorbing material.

As shown in Table 7, 27 °C aluminum plate and 28.4 °C absorbing material are selected as calibration sources for overall calibration. The physical figures are shown in Figure 24.

The emissivity of aluminum plate is estimated to be 0.26 and that of absorbing material is estimated to be 0.995 [17]. The temperature of aluminum plate and absorbing material are multiplied by their emissivity to get the emission temperature. Then, the emission temperature and corresponding voltage value are substituted into the overall calibration equation to obtain the overall calibration equation of the system.

1.213 = 7.02a + b(40)

1.255 = 28.258a + b(41)

From the above two equations, slope a ≈ 0.0019, intercept b ≈ 1.1999.

**Figure 25:**
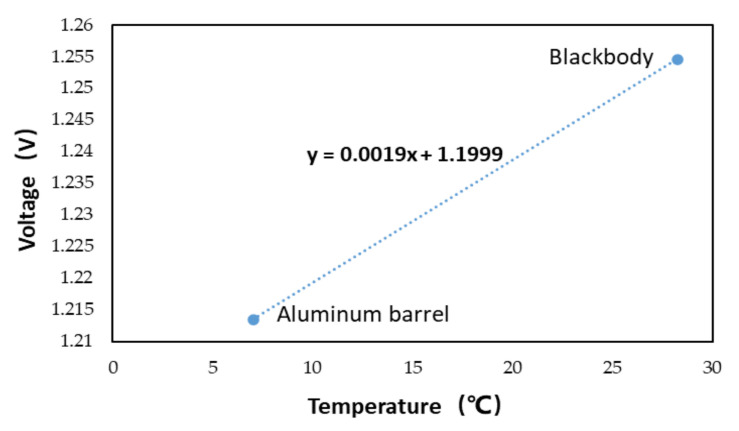
Overall calibration curve.

The overall calibration curve is shown in Figure 25:

After substituting the corrected voltage value of the verification data into the overall calibration curve, the overall calibration temperature is 28.03 °C. Compared with the result of step-by-step calibration, the overall calibration result is closer to the actual brightness temperature.

Due to the interference of electromagnetic environment, the loss of equipment components and the change of detection position will affect the calibration results, it is necessary to calibrate regularly.

#### 3.3.3. Water Temperature Inversion Algorithm in Near Field

According to the calibration equation, the brightness temperature of the measured object can be determined. Inversion algorithm determines the relationship between brightness temperature and physical temperature. In the actual measurement process, aluminum plate temperature $T_{al}$, antenna temperature $T_{\mathrm{an}}$ and feeder temperature $T_{\mathrm{fe}}$ will affect the measurement results. When the temperature change range is small, according to:

(42) $$T_{\mathrm{AP}}=T_{\mathrm{wa}}\times \varepsilon_{\mathrm{wa}}+T_{al}\times \varepsilon_{al}+T_{\mathrm{an}}\times K_{\mathrm{an}}+T_{\mathrm{fe}}\times K_{\mathrm{fe}}$$

$\varepsilon_{\mathrm{wa}}$ is emissivity of water,${\;\varepsilon }_{al}$ is emissivity of aluminum plate, $K_{\mathrm{an}}$ is contribution coefficient of antenna,$\;K_{\mathrm{fe}}$ is contribution coefficient of feeder.

The corresponding relationship between target brightness temperature $T_{\mathrm{AP}}$ and physical temperature $T_{\mathrm{wa}}$ can be determined.

The multiple linear regression algorithm and BP neural network algorithm are used to design the inversion algorithm respectively.

For the sample data with four parameters, the matrix representation of multiple linear regression is shown in Equation (43):

(43) $$T_{AP}(T_{0},{\;T}_{1},\;\cdots ,{\;T}_{4})=T\times \theta$$

$T_{AP}(T_{0},{\;T}_{1},\;\cdots ,{\;T}_{4})$ is brightness temperature, T is the temperature vector composed of water temperature, aluminum plate temperature, antenna temperature and feeder temperature, θ is the parameter vectors corresponding to different material temperatures.

The mean square error $J\left(\theta_{0},{\;\theta }_{1},\;\cdots ,{\;\theta }_{4}\right)$ is used as the loss function:

(44) $$J\left(\theta_{0},{\;\theta }_{1},\cdots ,\theta_{4}\right)={\left(T_{AP}-Y\right)}^{2}$$

Y is predicted temperature. The matrix form of the mean square error is shown in Equation (45):

(45) $$J\left(\theta \right)=\frac{1}{2}{\left(T_{AP}-Y\right)}^{T}\left(T_{AP}-Y\right)$$

The gradient descent method is used to calculate the value of θ until the loss function is minimized:

(46) $$\theta =\theta -\alpha T^{T}\left(\theta T-Y\right)$$

**Figure 26:**
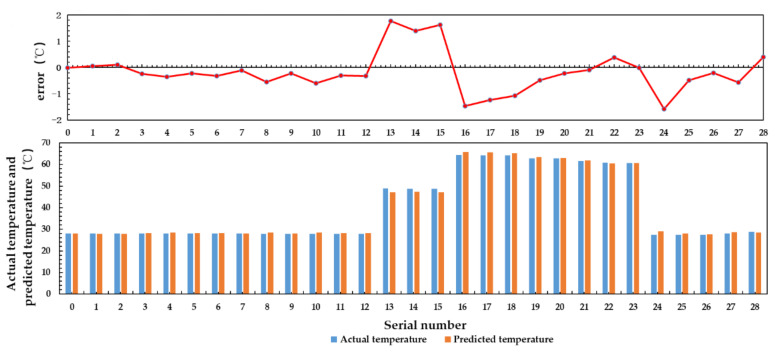
Inversion results of multiple linear regression.

In the multiple linear regression algorithm, 125 groups of sample data which the input are water temperature, aluminum plate temperature, antenna temperature, and feeder temperature, the output is actual water temperature are used to train the model parameters, and 29 groups of data are used to verify the model. The results are shown in Figure 26.

It can be seen from Figure 26, the predicted value obtained by using multiple linear regression algorithm is close to the actual value, and the mean square error is 0.607.

**Figure 27:**
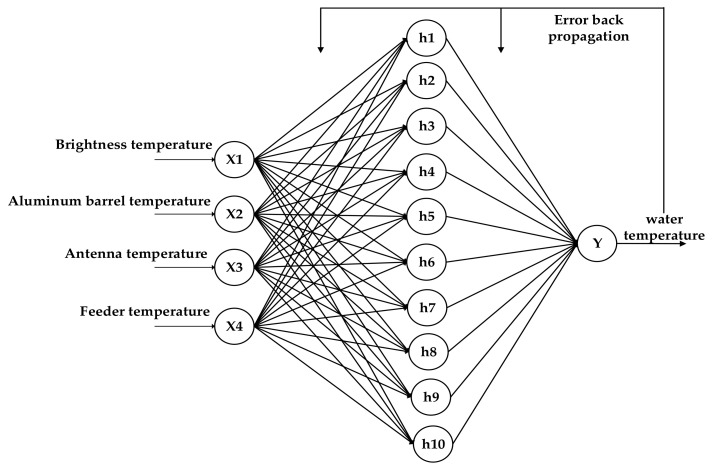
BP neural network model.

The BP neural network algorithm can be used in the water temperature inversion algorithm. BP neural network uses the steepest descent method to continuously adjust the weights and thresholds of the network through back propagation to minimize the sum of squares of the network errors. This process continues until the error of the network output is reduced to less than 0.1 °C or the number of learning times set in advance has reached. The BP neural network model is shown in Figure 27.

**Figure 28:**
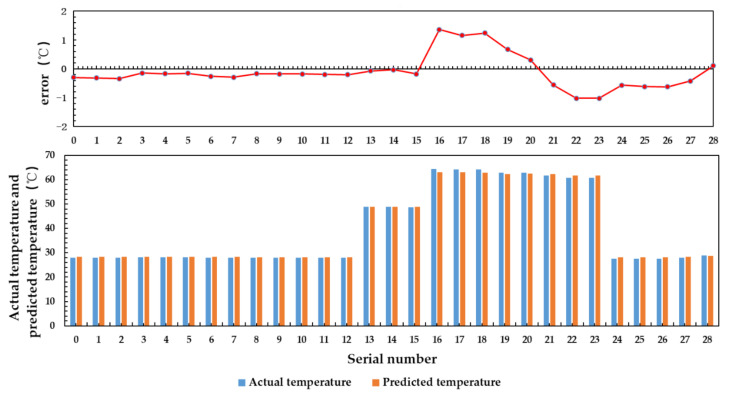
BP neural network inversion result.

The three-layer BP neural network is used to train 125 data sets for 10,000 rounds. The input variables of the data set are the brightness temperature of water, the aluminum plate temperature, the antenna temperature and the feeder temperature, and the output variable is the physical temperature of water. Twenty-nine groups of test data are used for verification. The result is shown in Figure 28.

The results show that the prediction accuracy of BP neural network for water temperature below 40 °C is higher than that for water temperature above 40 °C, and the mean square error of prediction is 0.334.

The deviation of the multiple linear regression algorithm and BP neural network algorithm is due to: (1) The number of samples is small, especially when the temperature is higher than 40 °C. The number of samples with temperature above 40 °C is 46 groups, which leads to a large deviation of data prediction when the temperature is higher than 40 °C. (2) The accuracy of temperature sensors for measuring aluminum plate temperature, antenna temperature, and feeder temperature is limited, which leads to low accuracy of the data used in the inversion algorithm. (3) The range of temperature change is large. The condition of Equation (42) is that the antenna coefficient and feeder coefficient remain unchanged when the temperature range is small. But the temperature range of the actual test data is large, so the antenna coefficient and feeder coefficient cannot be regarded as a constant.

Table 8 shows the prediction results of multiple linear regression algorithm and BP neural network algorithm. Compared with the results obtained under the conditions of large temperature range and uneven temperature distribution, the prediction accuracy is significantly improved after reducing the temperature range.

BP neural network algorithm can adaptively adjust the thresholds and weights to determine the optimal model structure. Compared with multiple linear regression, it has the advantages of flexibility and convenience. The inversion results show that the prediction accuracy of BP neural network is higher than that of multiple linear regression algorithm.

At present, the temperature measurement accuracy of infrared thermometer on the market is 0.2–0.4 °C when the measuring distance is 2–5 cm. The mean error of the interferometric microwave thermometer radiometer can reach 0.202 °C when the measurement distance is about 10 cm. In addition, the microwave radiometer can penetrate the clothing to measure the temperature of body and can also detect the temperature of subcutaneous tissue. This research is an institutional innovation for non-contact temperature measurement with microwave radiometer, which lays a foundation for the use of microwave radiometers for high-precision, non-contact body temperature measurement and subcutaneous tissue temperature measurement.

## 4. Discussion

The microwave radiometer designed in this paper uses a power divider to divide the measured signal into the same two channels, and then realizes the autocorrelation detection function through the quadrature demodulator. Compared with the traditional total power microwave radiometer, the detection sensitivity of the interferometric microwave thermometer radiometer increases by 3.48 times. In order to further improve the measurement accuracy and sensitivity of the interferometric microwave radiometer, the phase shifter is used to implement periodic 360° phase scanning to generate data samples, then the phase error, gain error, and offset error of the microwave radiometer are corrected by designing correction algorithm. The challenge now is that after the microwave radiometer works for a long time, the drift of phase and gain of radiometer lead to a decrease in accuracy and sensitivity. Therefore, it is necessary to keep the microwave radiometer at a constant temperature.

In order to solve the problem of temperature sensitivity decline caused by long-time unstable operation of receiver circuit, in step-by-step calibration, 75 and 50 Ω microwave loads are equivalent to “cold/hot” calibration source respectively to realize two-point calibration of radiometer receiver circuit. The overall calibration uses a conical array coated with absorbing material whose area is less than or equal to 100 ${\mathrm{cm}}^{2}$ to solve the problem of temperature sensitivity reduction caused by antenna and ambient temperature changes. A reasonable design of step-by-step/overall calibration fusion working mechanism can not only ensure the linear relationship between the brightness temperature and the output voltage of the microwave radiometer, but also can be used to test the sensitivity, linearity and stability of the microwave radiometer. In order to improve the measurement accuracy of microwave radiometer, both the step-by-step calibration source and the overall calibration source should be controlled with high-precision temperature control to solve the problem of temperature drift of the calibration source [30]. Currently, the challenge is that the temperature distribution of the cone array is uneven, which makes it difficult to accurately control the temperature of the calibration source.

In this paper, the gradient descent method of multiple linear regression algorithm is used to minimize the loss function and inverse the target temperature. BP neural network is used to adjust the weights and thresholds of the network by using the steepest descent method so as to minimize the sum of squares of the network errors. In the subsequent experiments, genetic algorithm (GA) and particle swarm optimization (PSO) will be used to optimize the weights and thresholds of each layer of BP neural network [31,32,33,34], which can improve the prediction accuracy of BP neural network. At present, the biggest challenge is the random electromagnetic interference in the environment and the random change of ambient temperature, which may lead to a great difference between the inversion result and the actual temperature of the target.

We have designed the prototype, and we will design it as a miniaturized instrument. In addition, the microwave radiometer is used to measure water temperature now, it will be used to measure body temperature in the future.

## 5. Conclusions

Due to microwave can achieve non-contact detections of human subcutaneous tissue temperature, it provides new non-contact detection approach to assist, treatment and rehabilitation of patients with severe diseases. In this paper, we study the key technology of the interferometric microwave thermometer radiometer.

(1) This paper innovatively uses 500–6000 MHz broadband quadrature demodulator to design the interferometric microwave thermometer radiometer. Compared with the traditional total power microwave radiometer, the detection sensitivity increases by 3.48 times. In addition, the detection bandwidth is 2 GHz and the circuit structure is simple. The maximum sensitivity of the microwave radiometer can reach 176.54 mV/dBm when the input point frequency signal power is 7 dBm. It can be concluded that the sensitivity of temperature measurement can reach ±0.40 °C/mV when the body temperature is 35–45 °C.
(2) The noise power is about −80.70 to −80.56 dBm when human body temperature ranges from 35 to 45 °C. In this paper, a multi-stage low noise amplifier is used in the RF front-end to make the total gain about 90 dBm, at this time, the microwave radiometer reaches the working point of the maximum sensitivity near 7 dBm, which ensures that the microwave radiometer has a higher detection sensitivity.
(3) At this time, the multi-correlator reaches the maximum sensitivity operating point near 7 dBm to ensure that the radiometer has a higher detection sensitivity.
(4) To reduce the influence of phase error, gain error and offset error of microwave radiometer on temperature measurement accuracy and sensitivity, the phase shift value of phase shifter is controlled to correct the system phase error and the gain error and offset error are corrected by using linear fitting algorithm. After correction, the phase error reduces from 40° to 1.4° and the voltage amplitude changes obviously when the temperature changes 0.1 °C.
(5) The microwave radiometer is calibrated by step-by-step calibration scheme and overall calibration scheme respectively. In the step-by-step calibration, the microwave radiometer is calibrated by microwave load with different resistance, the antenna is calibrated according to the antenna efficiency and the antenna physical temperature. In the overall calibration, aluminum plates and absorbing materials with different emissivity are selected to calibrate the whole system. The results show that the overall calibration result is consistent with the emissivity relationship of the measured object, and the step-by-step calibration can evaluate the change of calibration curve caused by the temperature rise of the microwave radiometer at any time.
(6) In order to evaluate the impacts of aluminum plate temperature, antenna temperature and feeder temperature on the output result, this paper accounts these factors in the inversion algorithm. Multiple linear regression algorithm and BP neural network algorithm are used to carry out inversion operation. The results show that the mean square error of multiple linear regression algorithm is 0.607 and that of BP neural network algorithm is 0.334. The inversion accuracy can be improved by reducing the temperature range. In the follow-up work, the accuracy of temperature measurement will be further improved by increasing the amount of sampling data and selecting better inversion algorithm.

The novel interferometric microwave thermometer designed in this paper can reverse the actual temperature from the input power, the temperature resolution is 0.4 K, the structure is simple and the bandwidth is wide. The research result of this project lay a research foundation for the realization of accurate, rapid and non-contact rapid detection device of human body temperature, which has certain practical value and social significance.

## Figures and Tables

**Table 1:** Mean output voltage of microwave radiometer when measuring water at 33–55 °C.

Date	6 August 2020															
Temperature (°C)	52.9	52.8	52.7	52.6	52.5	52.4	52.3	52.2	52.1	52	51.9	51.8	51.7	51.6	51.5	51.4
Voltage (V)	1.266	1.265	1.261	1.260	1.256	1.253	1.252	1.249	1.249	1.247	1.244	1.242	1.239	1.237	1.236	1.236
Date	8 August 2020															
Temperature (°C)	53.1	53	52.9	52.8	52.7	52.6	52.5	52.4	52.3	52.2	52.1	52	51.9	51.8	51.7	51.6
Voltage (V)	1.297	1.293	1.292	1.290	1.283	1.285	1.282	1.282	1.278	1.276	1.274	1.275	1.272	1.268	1.267	1.266
Date	19 August 2020															
Temperature (°C)	41	40.9	40.7	40.6	40.5	40.3	40.2	40	39.9	39.8	39.7	39.6	39.5			
Voltage (V)	1.290	1.290	1.286	1.287	1.284	1.280	1.278	1.278	1.279	1.275	1.276	1.276	1.273			
Date	20 August 2020															
Temperature (°C)	34.6	34.5	34.4	34.3	34.2	34.1	34	33.9								
Voltage (V)	1.293	1.290	1.286	1.284	1.283	1.280	1.278	1.280								

**Table 2:** Relationship between physical temperature and brightness temperature of aluminum plate.

	6 August 2020		13 August 2020	
	Physical Temperature	Brightness Temperature	Physical temperature	Brightness temperature
Aluminum plate	28.8 °C	7.488 °C	27 °C	7.02 °C

**Table 3:** The comparison of temperature resolution of different types of microwave radiometers.

Different Types of Microwave Radiometers	Temperature Resolution (K)
Total power microwave radiometer [6]	0.62
Dicke microwave radiometer [5]	0.11
The one-dimensional synthetic aperture microwave radiometer [25]	0.7106
Ka-band direct detection radiometer [26]	0.41
W-band direct detection radiometer [27]	0.5
Dicke microwave radiometer [28]	Aperiodic calibration 1.35 Periodic calibration 0.62 Thermostat 0.17
Ka-band AC radiometer [29]	0.47
Interferometric microwave radiometer designed in this paper	0.4

**Table 4:** Voltage and phase values of some sampled data.

Temperature (°C)	Voltage (V)	Phase (°)
38	1.286	49.068
37.9	1.257	50.377
37.8	1.233	51.242
37.7	1.220	51.468
37.6	1.212	51.711
37.5	1.203	51.824

**Table 5:** Voltage and phase error after correcting phase error, gain error and offset error.

Temperature (°C)	Voltage (V)	Phase error (°)
38	1.279	1.131
37.9	1.260	1.317
37.8	1.237	1.433
37.7	1.219	1.458
37.6	1.209	1.489
37.5	1.196	1.500

**Table 6:** Validation data (absorbing materials at 28.3 °C).

Measured Object	Temperature (°C)	Emissivity	Emission Temperature	Corrected Voltage (V)
Absorbing materials	28.3	0.995	28.1585	1.254

**Table 7:** Data of aluminum barrel and absorbing material for overall calibration.

Measured Object	Temperature (°C)	Emissivity	Emission Temperature	Corrected Voltage (V)
Aluminum plate	27	0.26	7.02	1.213
Absorbing material	28.4	0.995	28.258	1.255

**Table 8:** The prediction results of multiple linear regression algorithm and BP neural network algorithm.

	Large Temperature Range (27.5–64.5 °C)				Small Temperature Range (27.5–28.9 °C)			
	Mean square error	Mean Error	Max error	Mini error	Mean square error	Mean error	Max error	Mini error
Multiple linear regression algorithm	0.607	0.759	1.777	0.018	0.070	0.223	0.508	0.007
BP neural network algorithm	0.334	0.575	1.358	0.033	0.059	0.202	0.525	0.006

## Data Availability

Not applicable.
